# Predicting intratumoral fluid pressure and liposome accumulation using physics informed deep learning

**DOI:** 10.1038/s41598-023-47988-8

**Published:** 2023-11-23

**Authors:** Cameron Meaney, Shawn Stapleton, Mohammad Kohandel

**Affiliations:** 1 Department of Applied Mathematics, University of Waterloo, Waterloo, Canada; 2 MD Anderson Cancer Center, Houston, TX USA; 3 Department of Radiology, University of Washington, Seattle, WA USA

**Keywords:** Cancer, Mathematics and computing

## Abstract

Liposome-based anticancer agents take advantage of the increased vascular permeability and transvascular pressure gradients for selective accumulation in tumors, a phenomenon known as the enhanced permeability and retention(EPR) effect. The EPR effect has motivated the clinical use of nano-therapeutics, with mixed results on treatment outcome. High interstitial fluid pressure (IFP) has been shown to limit liposome drug delivery to central tumour regions. Furthermore, high IFP is an independent prognostic biomarker for treatment efficacy in radiation therapy and chemotherapy for some solid cancers. Therefore, accurately measuring spatial liposome accumulation and IFP distribution within a solid tumour is crucial for optimal treatment planning. In this paper, we develop a model capable of predicting voxel-by-voxel intratumoral liposome accumulation and IFP using pre and post administration imaging. Our approach is based on physics informed machine learning, a novel technique combining machine learning and partial differential equations. through application to a set of mouse data and a set of synthetically-generated tumours, we show that our approach accurately predicts the spatial liposome accumulation and IFP for an individual tumour while relying on minimal information. This is an important result with applications for forecasting tumour progression and designing treatment.

## Introduction

A key obstacle in the efficacy of anticancer chemotherapeutics is the chaotic, inefficient vasculature which commonly plagues solid tumours^1^. This irregular tumour vasculature causes decreased nutrient delivery, increased hypoxia, impaired drainage, and notably, high interstitial fluid pressure (IFP)^2^. High IFP in tumours has been associated with cancer progression and resistance to both chemo- and radio-therapy^1^. Accordingly, the level of IFP present in tumour tissues is a relevant clinical factor for forecasting tumour progression and designing optimal treatments. Unfortunately, measuring the level of IFP *in vivo* directly is challenging, relying on invasive procedures which would make it disqualifying in a clinical setting. Motivation therefore exists for the development of noninvasive methods capable of predicting high IFP, especially quantitatively, in a patient-specific manner.

Fortunately, recent advancements in medical imaging and liposome technology have provided an avenue to accomplish this. Specifically, a phenomenon termed the enhanced permeability and retention (EPR) effect - similarly caused by the aforementioned irregular tumour vasculature - results in a selective accumulation of liposomes within tumour tissue over healthy tissue^3,4^. While this preferential accumulation hasn’t yet been leveraged into a significant increase in drug efficacy over standard of care, the phenomenon is well-documented and able to be observed through imaging^3,5,6^. This imaged accumulation can then be combined with sophisticated quantitative methods to derive estimates for intratumoral IFP.

An established mathematical model which has been used in many studies (^7–15^, for example) quantitatively links interstitial liposome accumulation to intratumoral IFP. Given the IFP, in addition to the tissue- and liposome-specific model parameters, a map of liposome accumulation could be predicted pre-administration by solving the mathematical model. However, given that IFP is difficult to accurately measure *in vivo*, it is more useful to consider the inverse of this problem: namely, given the intratumoral accumulation of liposomes post-administration, it should be theoretically possible to derive the underlying IFP which resulted in that spatial distribution of liposome accumulation. Using standard mathematical techniques, this inverse problem is quite challenging to solve. However, a novel deep learning technique called physics-informed neural networks (PINNs) is well-suited to handle problems of this nature. PINNs are a type of neural network which incorporates data from a mathematical model into network optimization^16,17^. In this case, data from liposome accumulation imaging can be used in combination with the established mathematical model to estimate the IFP in that tissue. Spatially varying predictions of IFP for an individual patient could then theoretically be used in predicting disease progression, optimizing disease therapy, or evaluating treatment response.

Estimation of patient-specific IFP using noninvasive methodologies has been the aim of several previous studies. Bhandari et al conducted a series of investigations that leveraged magnetic resonance imaging (MRI) data from brain tumours. In their initial study^10^, they employed partial differential equations (PDEs) to solve for pressure distributions directly, utilizing parameter values for normal and tumour tissues from the literature. These pressure distributions were then used to predict the transport of cancer drug-carrying liposomes, deploying equations similar to those applied in this study. In a subsequent study^9^, Bhandari et al incorporated variable factors into their modelling, such as heterogeneous vasculature, theoretically enabling patient-specific estimations of IFP. In another work from the same group^8^, the authors extended their approach by solving for pressure using PDEs, and using the derived pressure values to predict the transport of various cancer chemotherapeutics. This was done by selecting tissue-specific parameters for normal and tumour tissues, substituting values for drug-specific parameters specific for each chemotherapeutic agent, and solving the equations to obtain concentration maps. Further advancements were made in^7^, where dynamic contrast-enhanced imaging was utilized to estimate patient-specific parameter values, enabling predictions of IFP and drug distribution for individual patients. Soltani et al^12^ introduced the concept of angiogenesis into the model and, similar to previous studies, and solved for pressure using PDEs with fixed parameter values. Liu et al.^11^ also applied MRI data to predict IFP by extracting key tumour measurements and using them to derive parameter values that informed the PDE for pressure. In a larger study, Swinburne et al.^15^ employed the PDE model for pressure and transport, applying it to a large dataset of 41 brain tumour patients. The study generated voxel-by-voxel estimates of pressure as well as mean pressure values for each tumour. In Hompland et al^18^, dynamic contrast-enhanced MRI was used with contrast agents of different weights was performed to find associations between the weight of the agent, the volume transfer constant, and the IFP. They found that for low weight contrast agents (< 3.5 kDa in their study), the volume transfer constant of the agent is associated with tumour IFP.

Importantly however, each of these works solved the forward problem, first selecting or estimating parameter values, then solving for pressure and subsequently drug distribution. None of them solved the inverse problem, beginning with drug distribution and using it to derive pressure, as we do here, which could lead to more specific and accurate IFP predictions. In Stapleton et al.^19,20^ however, the authors did attempt to address this inverse problem. They employed a series of CT scans to determine bulk liposome accumulation at various time points. Then using a fitting process based on the complete time series of bulk accumulation, they made predictions of the IFP. However, this method relied on the availability of a series of images, which is not typically feasible in clinical practice.

In Stapleton et al^19,20^ however, they attempted to solve this inverse problem. They used a series of CT scans to derive the liposome accumulation at a series of times. Then, through a fitting process using the full time series of bulk accumulation, they were able to make predictions on the IFP. However, this method relied on a series of images, which is typically not available in clinical practice.

**Figure 1:**
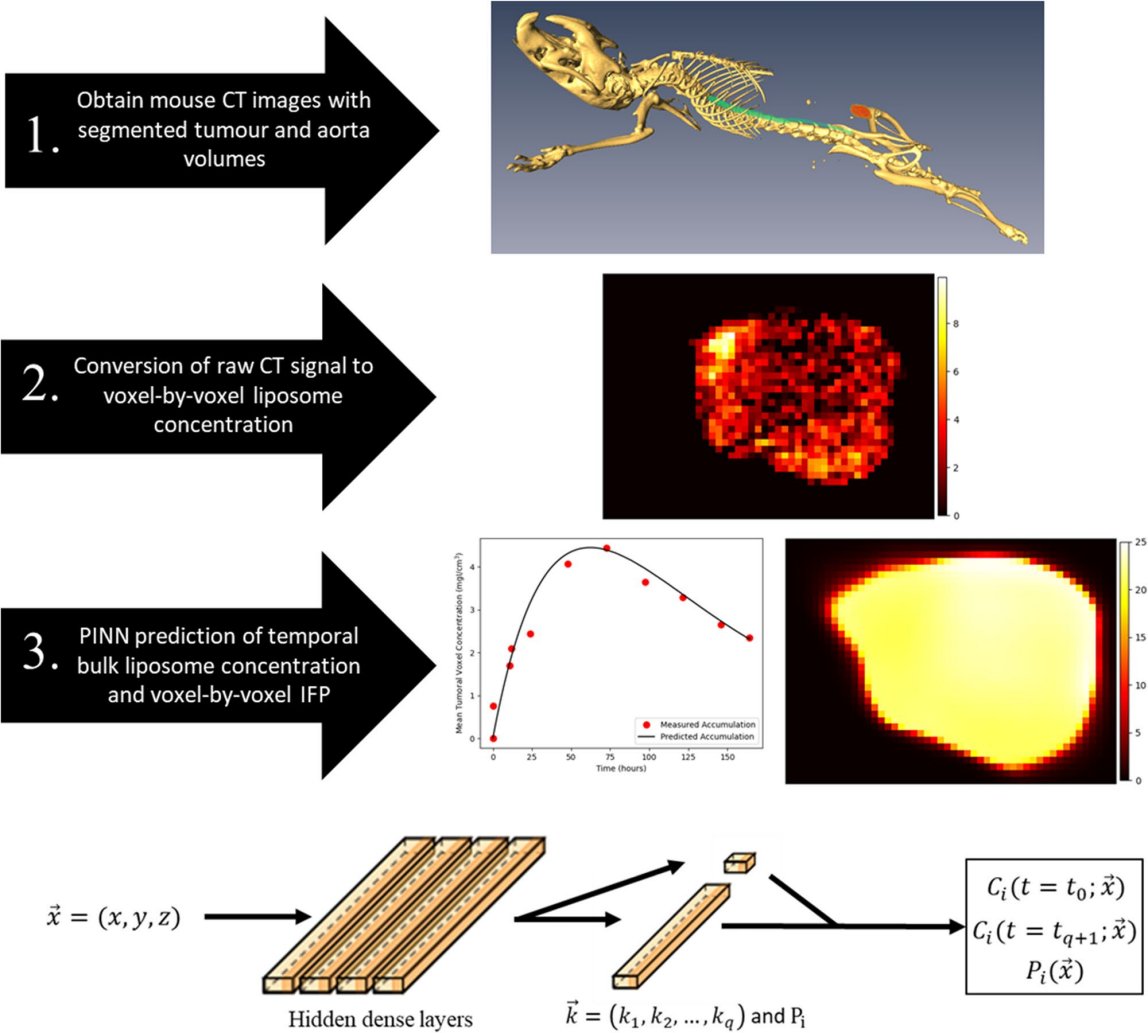
Diagram illustrating the workflow of our deep learning model. Following acquisition of CT imaging of segmented xenograft mouse tumours, the data is first cropped and converted to voxelwise liposome concentration at the final imaging time point. Then, our deep learning algorithm uses this data to predict the IFP and liposome accumulation curve. For our dataset, the predictions for liposome accumulation can be compared to additional measured liposome accumulation datapoints obtained through imaging, and the prediction for IFP can be compared to measured IFP using a wick-in-needle measurement. On the bottom, a visual representation of our deep learning model is shown. The spatial coordinates of each voxel are input to the network which consists of 4 dense layers each with 50 nodes, followed by a final dense layer on $q+1$ nodes. The outputs of this final layer are the predictions of intermediate liposome concentration at each time and pressure at the inputted voxel. These values are passed to the PDE model to compute the initial and final measured liposome accumulation maps derived from imaging, which are used to compute the loss and update the network.

**Figure 2:**
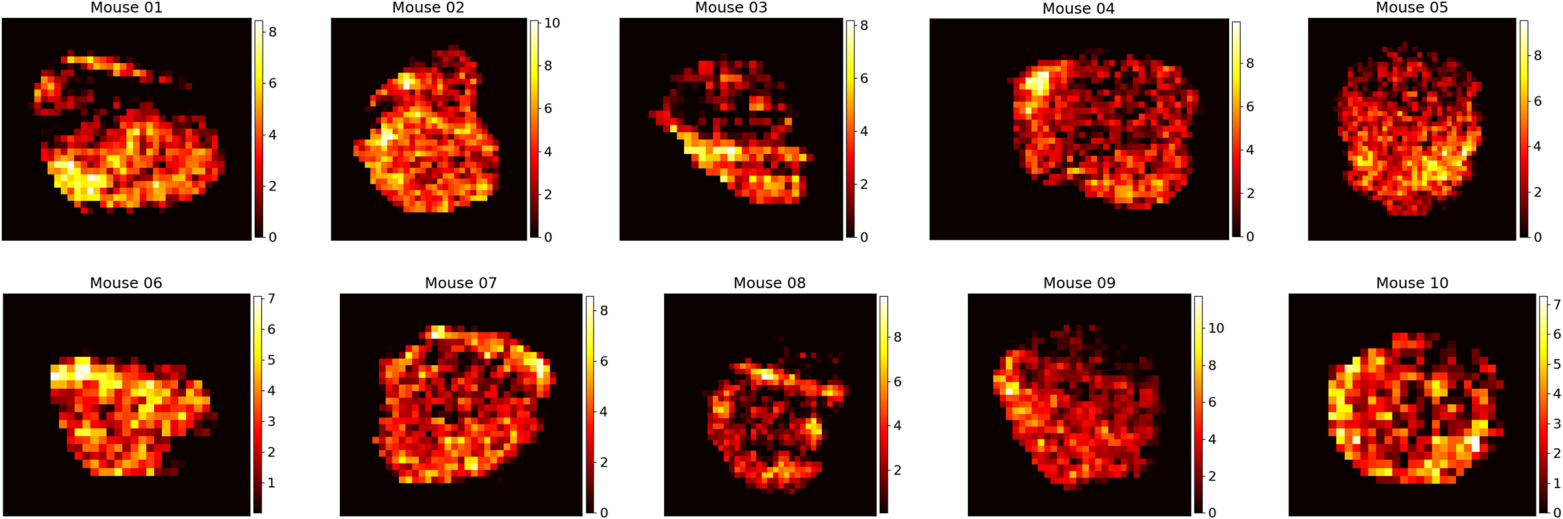
Final time spatial liposome accumulation maps for each of the 10 mice considered in our study. Raw CT signal in HU is converted to liposome accumulation using the above preprocessing procedure. Colour bar values are in units of mgI/cm$^3$. Though the full tumour concentration image is in 3D, a representative vertical slice approximately half way through the tumour core is shown here. These distributions are used as known data for the deep learning model and are compared to the network predictions to calculate the loss value during training.

**Figure 3:**
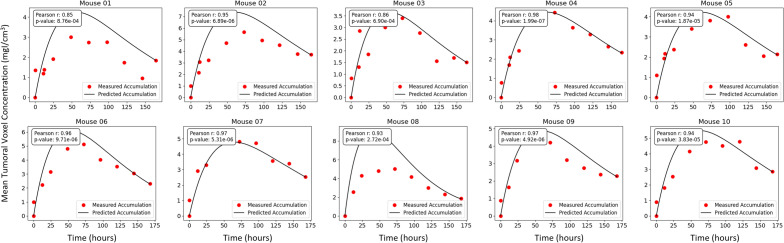
Comparison of the measured (red points) and predicted (black line) accumulation of liposomes in the tumour region for each of the 10 mice included in our study. Mean tumoral voxel concentration is calculated by taking the average concentration of all voxels in the labelled tumour volume at each time step.

In this paper, we develop a specialized deep learning model, based on physics informed machine learning, which is capable of predicting the spatiotemporal accumulation of liposomes and spatial IFP *in vivo* from a single image of liposome accumulation. The key idea behind physics informed machine learning is that established mathematical models governing a process of interest can be incorporated into the optimization of a neural network, allowing for faster and more accurate convergence while relying on less data. In order to make predictions, our model requires imaging of the spatial liposome accumulation within the tumour at a time post-administration as well as estimates of the parameters present in the mathematical model, which could be estimated in a patient-specific manner or taken from the literature as done in the previous studies mentioned above. Our model pipeline can be seen in Fig. 1. Our approach is an improvement over methods in previous studies, particularly^19,20^ which required a full time series of liposome accumulation imaging in order to perform curve fitting. Furthermore, our approach makes predictions of spatial IFP and liposome accumulation simultaneously, in contrast to previous methods which used temporal liposome accumulation to predict IFP. This makes our work relevant for treatment planning and monitoring, as well as provide a noninvasive measure of IFP as a prognostic biomarker. We apply our method to an animal dataset from a previously published liposome accumulation study^13^. In the materials and methods section below, we explain the data acquisition and preprocessing, as well as describe the key concepts and steps in our method including the PDE model and deep learning model used to make predictions of pressure. In the results section, we show the IFP predictions made by our model on the animal dataset and compare to the measured IFP to assess our model accuracy. We also conduct a sensitivity analysis on our model by applying it to a series of synthetically generated tumours and liposome accumulation maps. In the conclusion, we summarize the paper and discuss limitations and directions for future research.

## Methods

### Mathematical model

The key concept of PINNs, which we employ in the design of our deep learning model, is to incorporate information from a governing mathematical model into the training of the neural network through the loss function. In our work, we rely on a partial differential equation (PDE) model to relate liposome transport and IFP within tumour tissue, which is given by equation (1). This PDE has been used to model the transport of nanoparticles in tumour tissue in several previous works (^7–15^, for example), many of which provide a detailed derivation of the equation itself.

1 $$\begin{aligned} \frac{\partial C_i(\vec {x},t)}{\partial t} = \frac{L_pS}{V} \left( P_v - P_i(\vec {x}) \right) \left( 1 - \sigma \right) C_p(t) + \nabla \cdot \left( fKC_i(\vec {x},t) \nabla P_i(\vec {x}) \right) - k_d C_i \end{aligned}$$

In this model, $C_i(\vec {x},t)$ and $C_p(t)$ are the concentrations of liposomes in the interstitium and plasma respectively. Note that the interstitial concentration is allowed to vary throughout the tumour both spatially and temporally, whereas the plasma concentration is assumed to vary only temporally. In other words, the plasma concentration of liposomes is assumed to be equal in each voxel in the tumour image, and only change its concentration over time. In healthy tissues, pressures are tightly regulated to ensure homeostasis, though in tumours, these pressures can become unregulated. The first term of the right hand side of equation (1) describes fluid flow from the plasma into the interstitium, which is governed primarily by the difference between the IFP, $P_i(\vec {x})$, and the microvascular pressure, $P_v$. Here the microvascular pressure is assumed to be constant in space and time. This fluid motion is also dictated by the vascular hydraulic conductivity, $L_p$, the vessel surface area per unit tissue volume, *S*/*V*. The reflection coefficient, $\sigma$, quantifies the fraction of molecules unable to travel via fluid flow due to particle-specific qualities. Since $\frac{L_p S}{V}$ always appears as a grouping, we consider it as a single parameter throughout our work and refer to it as the transvascular fluid exchange rate. Within the interstitial space, nanoparticles undergo both a convective and diffusive process. The second term on the right hand side of equation (1) describes the convection of nanoparticles from areas of high IFP to areas of low IFP. This process also depends on two parameters: the retardation coefficient, *f*, and the interstitial hydraulic conductivity, *K*. While in the interstitium, nanotherapeutics also undergo elimination, either through natural degredation, lymphatic , or cellular uptake. We term this grouped effect the elimination rate, and denote it $k_d$. In the above, $\nabla$ is the gradient operator which operates in as many spatial dimensions as the problem contains. Additionally, note that $\frac{L_p S}{V}$, $\sigma$, *f*, *K*, and $k_d$ are all allowed to vary spatially throughout the domain of interest. In particular, it is often assumed that each parameter takes on two values: one within tumour tissue and one within healthy tissue. See Table 1 below for a summary of the parameters appearing in the PDE model. Note that some studies which use this PDE model for liposome transport also assume liposome diffusion in the interstitium, which is incorporated into the model by adding a term of the form $D \nabla ^2 C_i$ where *D* is the liposome diffusivity. Though it is argued in many studies that the effect of diffusion is weak compared to the effect of convection for large molecules such as liposomes^14^. In our case, since the study from which our data comes did not include diffusivity, we omit it when operating on the mouse data as well^13^. Importantly We have included the diffusivity in the sensitivity analysis section below however, since many relevant studies in the literature do include it, and it is a straightforward addition.

**Table 1:** Summary of parameters included in the PDE model (1), which has been used in many previous studies. Note that the diffusivity is not included in our model when operating on our mouse dataset since the work this data originates from^13^, and to which we compare our predictions to, did not include it. The diffusivity has been included in the sensitivity analysis however because it is included in many other studies.

Symbol	Name	Units	Tissue- vs. Particle- Specific
$\frac{L_p S}{V}$	Transvascular Fluid Exchange Rate	1/mmHg*s	Particle
$P_v$	Microvascular Fluid Pressure	mmHg	Tissue
$\sigma$	Reflection Coefficient	1	Particle
*K*	Interstitial Hydraulic Conductivity	cm$^2$/mmHg*s	Particle
*f*	Retardation Coefficient	1	Tissue
$k_d$	Elimination Rate	1/s	Tissue
*D*	Diffusivity	cm$^2$/s	Particle

**Table 2:** Parameter values used for the mouse simulations in our study. All parameter values were taken from Stapleton et al^13^. Note that each parameter other than $P_v$ is given by a step function, with one value in the normal tissue and another value in the tumour tissue. The subscript *N* denotes the value in the normal region and the subscript *T* denotes to the value in the tumour region.

Parameter	Units	Value
$\left( \frac{L_PS }{V} \right) _N$	1/mmHg$\cdot$s	2.52e-6
$\left( \frac{L_PS }{V} \right) _T$	1/mmHg$\cdot$s	2.6e-6
$\sigma _N$	1	1.0
$\sigma _T$	1	0.2
$f_N$	1	1.0
$f_T$	1	0.5
$K_N$	cm$^2$/mmHg$\cdot$s	8.53e-8
$K_T$	cm$^2$/mmHg$\cdot$s	4.13e-8
$P_v$	mmHg	25
$k_{d_N}$	1/s	1.65e-6
$k_{d_T}$	1/s	5.10e-6

### Physics-informed deep learning model

Viewed purely as a mathematical problem, it is theoretically possible to derive the underlying IFP, $P_i(\vec {x})$, from the liposome accumulation, $C_i(\vec {x}, t)$, using the liposome transport model(equation (1)). In practice however, information of $C_i(\vec {x}, t)$ is obtained via imaging, meaning that the full continuous solution is not known, but rather data is typically only available at a number of discrete times; in this case, two of them. The mathematical problem faced then, is to find the best fit approximation of $P_i(\vec {x})$ such that equation (1) is satisfied and $C_i(\vec {x}, t_1) = u_1(\vec {x})$ and $C_i(\vec {x}, t_2) = u_2(\vec {x})$, where $t_1<t_2$ and the profiles $u_1(\vec {x})$ and $u_2(\vec {x})$ are the known liposome accumulation maps obtained through imaging. This can mathematically be viewed as a function inference problem in which we seek to minimize the error in the known data points as well as satisfy the governing PDE. Hence, it is well-suited for the application of PINNs.

**Figure 4:**
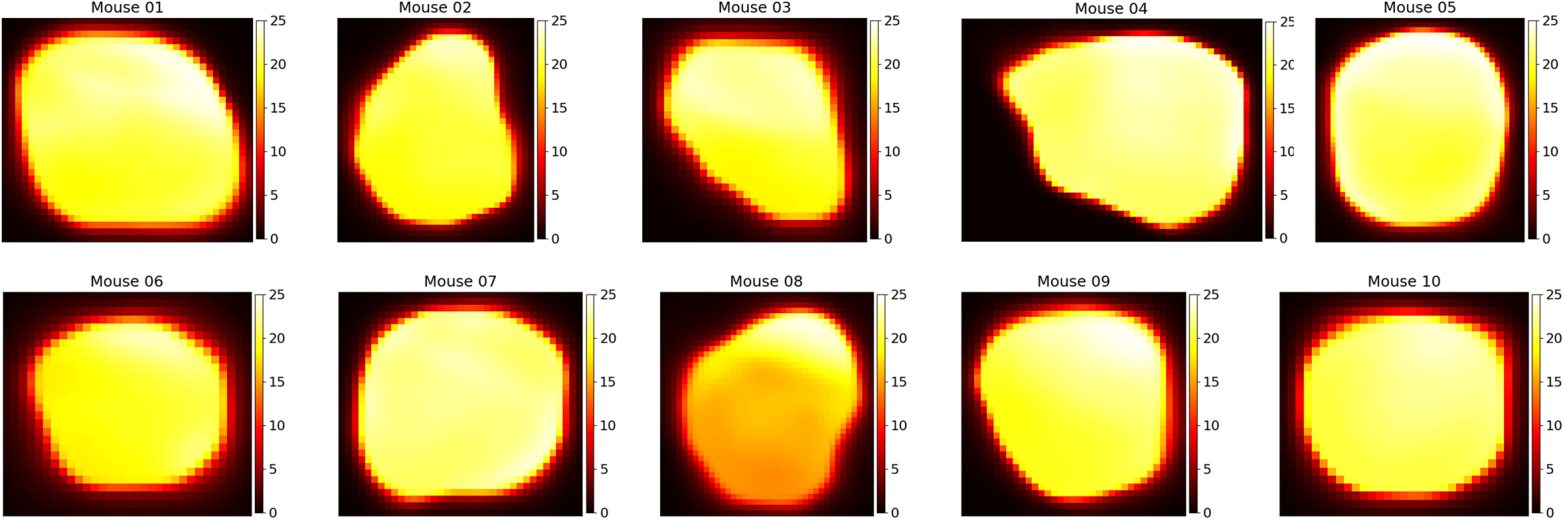
Spatial distributions of intratumoral IFP for each of the 10 mice considered in our study. Colour bar values are in units of mmHg - note that $P_v=25$ mmHg is an upper bound for IFP ($P_i$. Though the full tumour pressure image is in 3D, a representative vertical slice approximately half way through the tumour core is shown here. These distributions are used in Eq. 1 to calculate the loss value during training.

**Figure 5:**
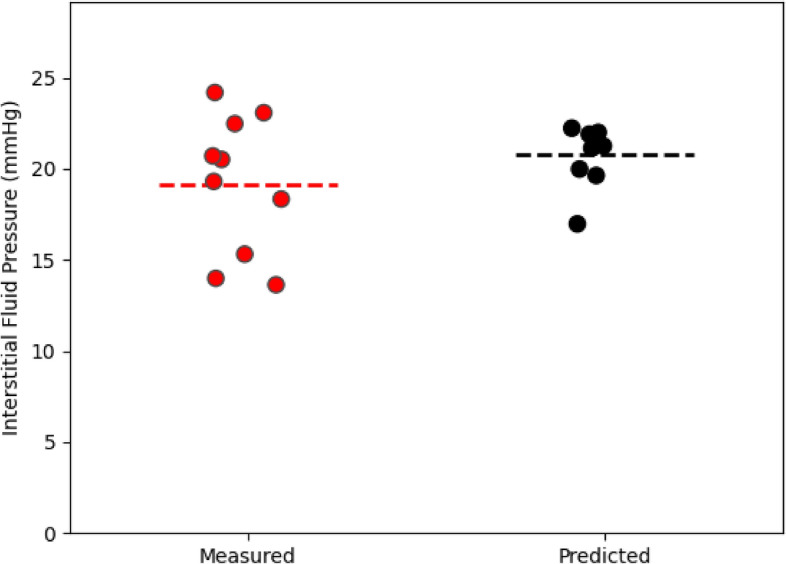
Comparison of the measured (red points) and predicted (black points) intratumoral IFP. Measured IFP was obtained in^13^ who took an average over 3 or 4 wick-in-needle pressure measurements in the tumour volume. Predicted IFP was obtained by taking the mean over all voxels in the tumour region. Horizontal dotted lines are placed at the mean of each case. For the measured average IFP values, the minimum, maximum, and mean values were 13.63 mmHg, 24.18 mmHg, and 19.15 mmHg whereas for the predicted IFP average IFP values, they were 16.97 mmHg, 22.22 mmHg, and 20.77 mmHg. The standrad error of the mean is 1.20 mmHg for the measured IFP and 0.50 mmHg for the predicted pressure.

**Figure 6:**
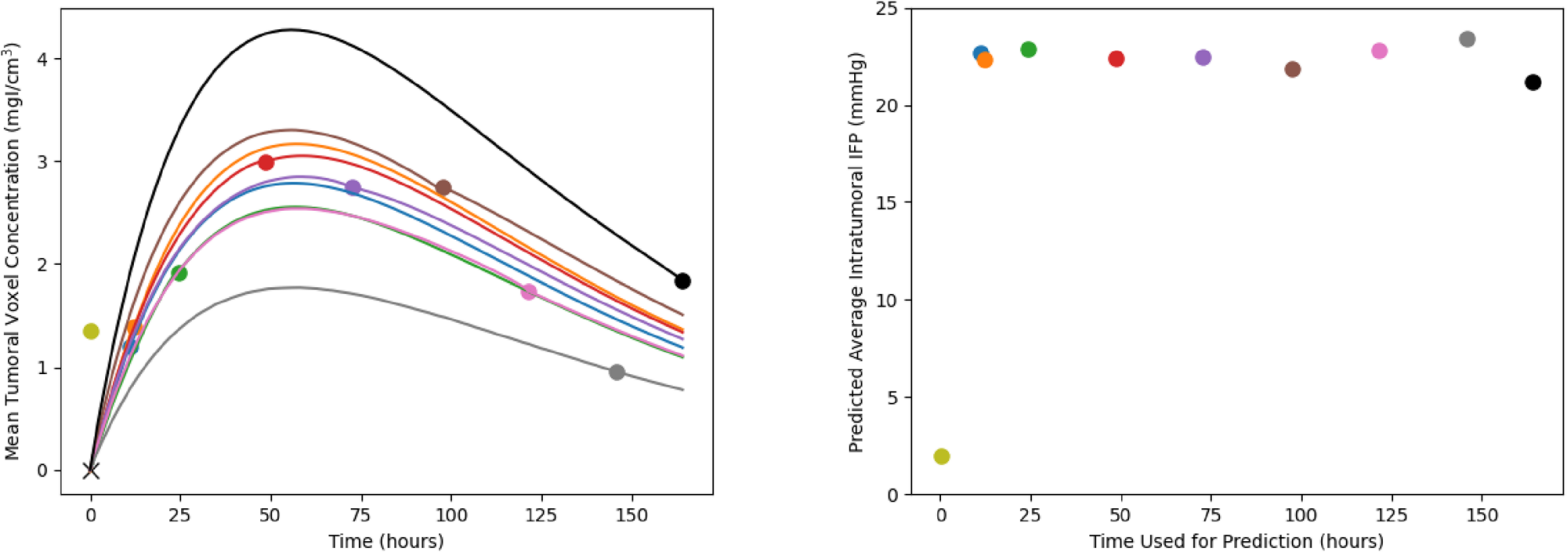
Differences in deep learning model predictions based on which final time point is used as input. **Left**: the different mean liposome concentrations throughout the tumour. The red points are the measured accumulations calculated from imaging and the black lines are the predictions of the network. Each line goes through exactly two known points (the point marked with an ‘x’ at time and concentration zero, and the dot with matching colour to the line). Note that the prediction using the first time point (at 10 minutes) is omitted from the graph as it has a high maximum which makes the rest of the graph difficult to interpret. **Right**: the mean intratumoral IFP as a function of the time point used for predictions (with colour matched to the colour on the left). Notice that, with the exception of the first time point at 10 minutes, the mean intratumoral pressure remains relatively steady while changing the known data.

**Figure 7:**
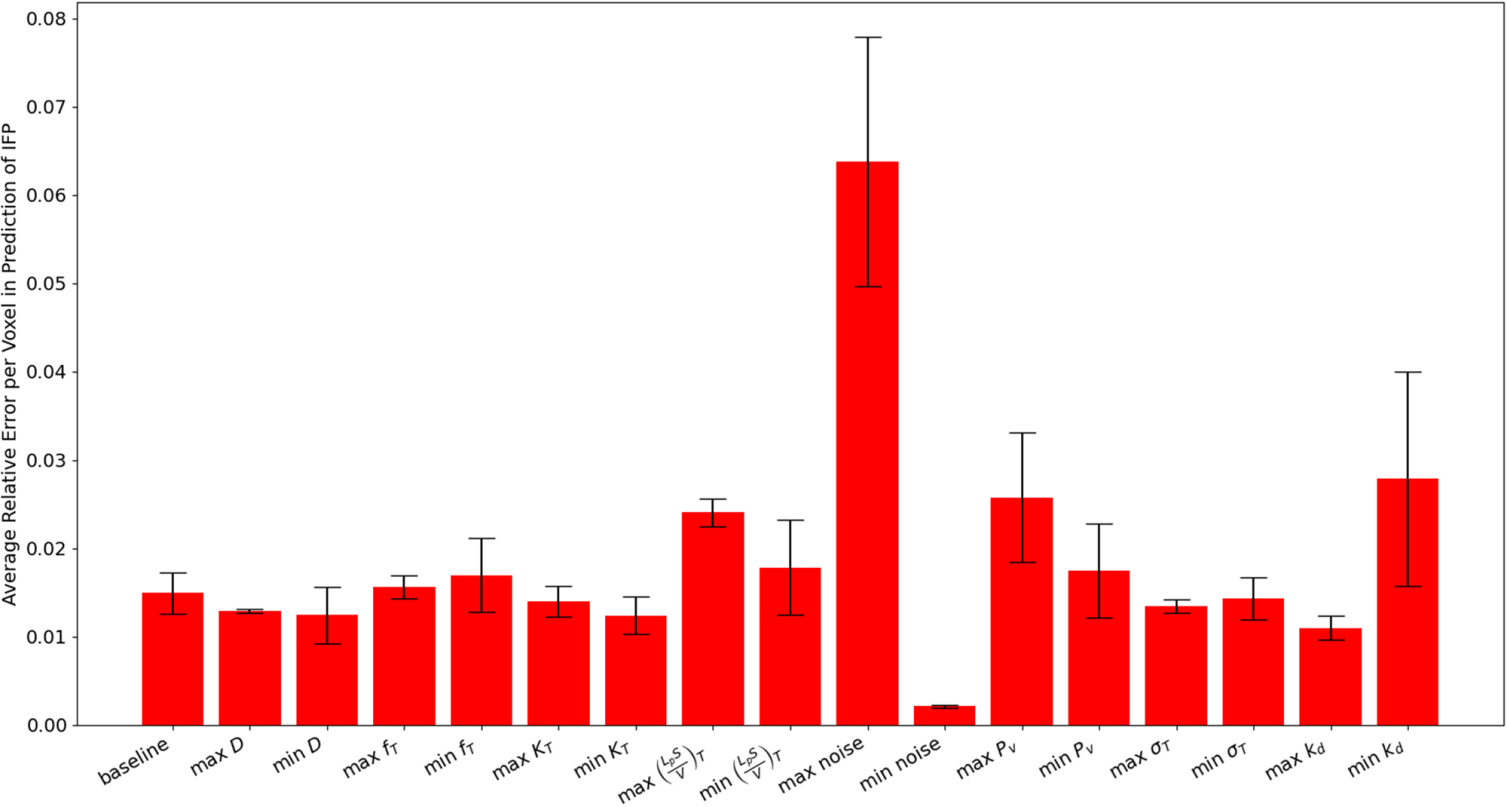
Average relative mean squared error in the prediction of IFP ($P_i$) for synthetic tumours generated using particular parameter sets. The columns denote either the set of baseline parameters in Table 3 or the set of baseline parameters with one of them made either the maximum or minimum of a chosen range, also given in Table 3. The height of each bar represents the average error over the 1000 bootstrap samples and the error bounds represent the 95% confidence intervals.

Consider the time discretization of the liposome transport model according to an implicit Runge-Kutta scheme with *q* stages over the interval $[t_1,t_2]$. We denote the Butcher tableau parameters $a_{nm}$, $b_n$, and $c_n$, and write the intermediate Runge-Kutta stages as $k_n(\vec {x})$ where $n \in [1,q]$. Note that each $k_n(\vec {x})$ is a prediction of $C_i(\vec {x},t)$ at an intermediate time between $t_1$ and $t_2$ corresponding to the Runge-Kutta parameter $c_n$. We can write this numerical scheme as

2 $$\begin{aligned} k_n(\vec {x})&= u_1(\vec {x}) + \Delta t \sum _{m=1}^q a_{nm} g \left( k_m(\vec {x}), t_1 + c_m \Delta t \right) \end{aligned}$$

3 $$\begin{aligned} u_2(\vec {x})&= u_1(\vec {x}) + \Delta t \sum _{m=1}^q b_m g \left( k_m(\vec {x}), t_1 + c_m \Delta t \right) \end{aligned}$$

where $u_1(\vec {x})$ and $u_2(\vec {x})$ are the measured liposome accumulation maps at $t_1$ and $t_2$ respectively, $\Delta t = t_2 - t_1$, and $g(\cdot , \cdot )$ is the right hand side of equation (1) written as $\frac{\partial C_i}{\partial t} = g(C_i,t)$. When typically using a Runge-Kutta scheme, an implicit matrix equation is solved for all *q* of the $k_n(\vec {x})$ profiles, which can then be used to calculate the $u_2(\vec {x})$ from $u_1(\vec {x})$. This process would be performed iteratively over many time steps to estimate the solution at later times. In this case however, both $u_1(\vec {x})$ and $u_2(\vec {x})$ are known, but the function $g(\cdot , \cdot )$ cannot be computed since the IFP, $P_i(\vec {x})$, is unknown. Instead, we can invert this numerical scheme to write the known initial and final liposome accumulation maps as

4 $$\begin{aligned} u_1(\vec {x})&= k_n(\vec {x}) - \Delta t \sum _{m=1}^q a_{nm} g \left( k_m(\vec {x}), t_1 + c_m \Delta t \right) \end{aligned}$$

5 $$\begin{aligned} u_2(\vec {x})&= k_n(\vec {x}) + \Delta t \sum _{m=1}^q (b_m - a_{nm}) g \left( k_m(\vec {x}), t_1 + c_m \Delta t \right) . \end{aligned}$$

This formulation of the Runge-Kutta scheme allows the known data (liposome accumulation snapshots) and the governing model (Eq. (1)) to be included in the loss function of our network training, as explained below.

We create a neural network which takes the spatial position of a voxel $\vec {x}$ as input, and outputs the liposome accumulation in that voxel at each intermediate time in the Runge-Kutta scheme, $(k_1(\vec {x}),...k_q(\vec {x}))$ (hence, 3 inputs and *q* outputs). These *q* intermediate stages can then be used to compute estimations for the initial and final liposome accumulation snapshots, $C_i(\vec {x},t_1)$ and $C_i(\vec {x},t_2)$, using Eqs. (4) and (5) which can be compared to the known liposome accumulation maps derived from imaging, $u_1(\vec {x})$ and $u_2(\vec {x})$. We choose a mean squared error metric to quantify the differences, and hence, the loss function used in network training is written as

6 $$\begin{aligned} MSE = \frac{1}{N} \sum _{\vec {x}} \left[ C_i(\vec {x}, t_1) - u_1(\vec {x}) \right] ^2 + \left[ C_i(\vec {x}, t_2) - u_2(\vec {x}) \right] ^2 \end{aligned}$$

where *N* is the total number of voxels in the liposome accumulation image and the sum is computed over all voxels in the image.

While the above formulation allows the inclusion of all of the available information into the optimization, it does not address the problem of unknown pressure. Fortunately, a key capability of this style of network optimization is that quantities appearing in the PDE model can be estimated during the training of the network. In the same way that the network optimizes the values of its weights, it can iteratively optimize the parameters or functions appearing in the model to find which values best fit the known data. In the original papers outlining PINNs, Raissi et al^16,17^ showed how PINNs could accurately estimate the values of unknown parameters in Burgers’ equation and identify pressure fields appearing in the Navier-Stokes equations. More recently, we used this technique in combination with brain tumour segmentation algorithms to characterize human glioblastoma multiforme by estimating patient-specific parameters appearing in a common model of brain tumour progression^21^. To make these estimations, a final custom layer is added to the neural network which calculates the loss using the PDE and unknown IFP. This custom loss layer can be backpropogated through to update the values of the unknown pressure in each voxel. In other words, this layer implements the PDE and loss function (Eq. (6)) by including an array for IFP, each voxel of which is an independent, trainable parameter which is optimized to the data and PDE throughout the network training. As the network trains, $u_1(\vec {x})$ and $u_2(\vec {x})$ converge to $C_i(\vec {x}, t_1)$ and $C_i(\vec {x}, t_2)$, and $P_i(\vec {x})$ should converge to the true pressure underlying the system.

One important note is that boundary conditions are not needed to train the network using the data and PDE. As explained in^16,17^, the boundary conditions are assumed to be present in the system which generated the data, and therefore, information on the boundary conditions is assumed to be present in the data itself. Therefore, there is no need to directly specify them in the optimization. It would be possible to add further terms to the loss function to quantify the satisfaction of particular boundary conditions to the network predictions - in much the same way that this is done for the PDE - but this would be challenging since defining the boundary on which these boundary conditions act is nontrivial in the context of complicated tumour geometries. Additionally, the boundary conditions that would typically be applied to the liposome transport model are continuity conditions at the tumour boundary and a zero-at-infinity condition, both of which are not straightforward additions to this form of discretized model.

### Mouse data collection and preprocessing

The mouse liposome accumulation maps used in this study were obtained as part of a previous investigation conducted by Stapleton et al^13^. In that study, 10 female SCID mice were injected with the MDA-231 human breast adenocarcinoma tumour cell line and allowed to grow until reaching a volume of 140 mm$^3$. A liposome-based CT contrast agent was then prepared and administered to all mice considered in the study. Liposome accumulation CT imaging was performed at various time points using a micro-CT system. To ensure consistency, a laser positioning system was used to place the mice in approximately the same orientation for successive scans, and manual rigid registration was performed after imaging. The tumour volume and descending aorta were then manually contoured on each CT data set.

A second set of mice were also used to measure intratumoral IFP. As the wick-in-needle pressure measurement method can disrupt the transport of liposomes, a separate set of mice was required in order to measure IFP. The intratumoral IFP was calculated by taking 3 or 4 measurements of IFP and finding the mean values. Importantly, the mice that were imaged and used to measure liposome accumulation were a different set of mice that were used for measuring IFP.

For each mouse, the average signal in the aorta volume was used to calculate the plasma concentration after scaling with a species-specific factor (50.1 HU/mgI$\cdot$cm$^3$) and hematocrit factor (0.5 unitless), as described in^14^. The resulting plasma concentration at each imaging time was used to fit a continuous plasma concentration function of the form $C_p(t) = ae^{-bt}$, which was then used in Eq. (1). Calculating the interstitial concentration of liposome is not as simple as scaling the CT signal. Specifically, each tissue voxel contains a vascular compartment with concentration $C_p$ and volume fraction $\epsilon _p$, a cellular compartment witch concentration $C_c$ and volume fraction $\epsilon _c$, and an interstitial compartment with concentration $C_i$ and volume fraction $\epsilon _i$. The total concentration can then be written as

7 $$\begin{aligned} C_{total} = \varepsilon _p C_p + \varepsilon _c C_c + \varepsilon _i C_i \end{aligned}$$

Based on the low fraction of endocytic cells observed experimentally by Stapleton et al^13^, we assume that the cellular concentration of liposome was negligible, and therefore that $C_c \approx 0$. This assumption was similarly made by Stapleton et al^13^ in their modelling. In addition, at early times, close to 100% of the injected liposomes remain in the plasma compartment, allowing us to make the assumption that $C_i \approx 0$. Using the measured value of $C_p$ from the aorta and the average voxel tissue concentration at an early time (10 minutes in this case), the interstitial volume fraction can be calculated. At future times, the interstitial concentration of liposomes can be solved for by using the same equation, but by measuring the full tumour concentration $C_{total}$ and the plasma concentration using the aorta signal $C_p$, and by using the previously calculated value for the interstitial volume fraction $\varepsilon _i$. This approach yields a voxel-by-voxel interstitial concentration of liposomes derived directly from CT imaging. Herein, we assume the values of $\varepsilon _i=0.30$ and $\varepsilon _p-0.03$ as calculated by Stapleton et al^13^.

After conversion to concentration, the tumour contour was used to crop the image to the tumour area with a small buffer and remove liposome signal outside of the tumour area. While it is not a perfect assumption since liposomes are expected to move slightly outside of the tumour area, we are focused on the magnitude and shape of the pressure profile inside the tumour, and thus ignore liposomes outside of its volume. Figure 2 shows representative cross sections of the resulting conversions for each mouse.

### Sensitivity analysis on synthetic tumours

In addition to the application of the model on liposome accumulation maps are synthetically generated using the liposome transport model ((1)) directly. In particular, an IFP profile, initial condition, tumour geometry, and set of parameters can be selected and used to solve equation (1) directly; then, the pressure identification deep learning model can be applied to the result. This serves two purposes. First, since the underlying IFP is known, we can compare the network predictions to the true pressure to assess the error, allowing us to showcase the model capabilities. Second, through generating many of these profiles using various PDE parameter values, we can assess areas where we expect the network to perform well and poorly. Though the mouse experiments above are performed in three spatial dimensions, we choose to perform the following sensitivity analysis in just one spatial dimension. This choice is made purely to reduce the computational expense involved: each increase in the spatial dimension of the problem increases the computation run time by a factor of the number of voxels in the dimension, which typically needs to be in the range of approximately 30 in order to obtain meaningful results. Importantly, this reduction in spatial dimension affects the code only in the encoding of equation (1) (partial derivatives only need to be calculated in one spatial dimension) and in the amount of data on which the network trains. All other parts of the network, including its architecture and training, are unchanged based on the spatial dimension of the problem.

To perform this sensitivity analysis, we assume a baseline of parameter values as given in Table 3. Then, using these parameters, the IFP is obtained from the PDE

8 $$\begin{aligned} \nabla ^2 P_i(x) = - \alpha (x)^2(P_e(x) - P_i(x)) \end{aligned}$$

which was originally derived by Baxter and Jain^22^. This equation is solved over an interval of length 10cm, equally partitioned into 200 units. The solving is accomplished with a finite element method with a zero-at-infinity boundary condition. To implement this computationally, a zero Dirichlet boundary condition is used and the computational domain is extended farther than necessary, which adds extra computational expense to the code solving, but is a better approximation of the infinite boundary condition. In the above equation, $\alpha (x)$ is given by

9 $$\begin{aligned} \alpha (x) = {\left\{ \begin{array}{ll} \sqrt{ \frac{1}{K_{T}} \left( \frac{L_p S}{V} \right) _{T}}, &{} \text {if }x \in \text { tumour} \\ \sqrt{ \frac{1}{K_{N}} \left( \frac{L_p S}{V} \right) _{N}}, &{} \text {if }x \notin \text { tumour} \end{array}\right. } \end{aligned}$$

where the individual parameters are given in Table 3. Similarly, we can write

10 $$\begin{aligned} P_e(x) = {\left\{ \begin{array}{ll} P_v, &{} \text {if }x \in \text { tumour} \\ 0, &{} \text {if }x \notin \text { tumour} \end{array}\right. } \end{aligned}$$

with $P_v$ also given in Table 3. In the above, we define the tumour area as an interval of diameter 2cm in the centre of the computational domain. Using this pressure, equation (1) is solved over the same spatial domain using a finite element method in space and a Crank-Nicolson time stepping algorithm. The solution is obtained over the time interval of [0, 200] hours using 150 equidistant time points. A zero flux boundary condition is applied to the PDE at the edges of the computational domain. This yields the function $C_i(x,t)$, from which the initial and final times can be selected as $C_i(x,t_1)$ and $C_i(x,t_2)$ and used as data for training of our deep learning model.

**Table 3:** Parameter values used in the sensitivity analysis.

Parameter	Units	Baseline value	Min value	Max value
*D*	cm$^2$/s	7.5e-7	0	7.5e-6
$f_T$	1	0.5	0.4	0.6
$K_T$	cm$^2$/mmHg*s	4.13e-9	1.0e-9	1.0e-6
$\left( \frac{L_PS }{V} \right) _T$	1/mmHg*s	2.6e-6	1.0e-7	1.0e-4
Noise	1	0.025	0	0.05
$P_v$	mmHg	25	5	50
$\sigma _T$	1	0.19	0	0.5
$k_d$	1/s	5.1e-6	1.0e-7	1.0e-5

The deep learning model is used to predict pressure for the base parameter set as well as the base parameter set with one parameter altered to be the minimum or maximum of a reasonable range. The baseline values are chosen to be the same as those estimated by Stapleton et al^13^ as explained above (apart from the noise, which is obviously not added to the mouse data), and the ranges are chosen to be purposefully broad to span a large subset of the parameter space. Note that we have included *D* as a parameter here even though we did not include it when making predictions on our mouse dataset. We include it here since many works which examine this or similar equations have included diffusion, and it is a simple addition. For each of these cases, the deep learning model is used with the synthetically generated liposome accumulation maps to derive an estimation for $P_i$, which can be compared to the $P_i$ obtained from solving equation (8). This error can then be quantified. In summary, the procedure for our sensitivity analysis is:

1. Select parameters by taking the baseline parameter set, and possibly altering one parameter
2. Solve equation (8) with the chosen parameters to obtain the IFP, $P_i(x)$.
3. Use the chosen parameters and calculated $P_i$ to solve equation (1) and obtain the liposome accumulation over time, $C_i(x,t)$.
4. Use the initial and final time distributions of $C_i(x,t)$ as known data in the deep learning model to estimate the best fit approximation of $P_i(x)$.
5. Compare the calculated and predicted $P_i(x)$ and report the relative mean squared error between the profiles.

### Neural network implementation

All simulations were implemented in python using TensorFlow 2.3.1. The number of intermediate Runge-Kutta stages was always chosen to be $q=100$. The network structure in all cases consisted of 4 fully connected layers with 50 nodes per layer, and all nodes utilized the built-in hyperbolic tangent activation function. The training uses a combination of an Adam optimizer^23^ (an extension of the stochastic gradient descent algorithm) and an L-BFGS optimizer^24^ (a limited memory version of the BFGS operator). The PDE and data were nondimensionalized using the variable scaling factors of $\tilde{t} = t * \left( P_v \mu _T \right)$, $\tilde{x} = x * \left( \sqrt{\frac{\mu _T }{\eta _T}} \right)$, and $\tilde{P} = P/P_v$. Additionally, prior to training, the network inputs were normalized to the interval [-1,1] and the network outputs were normalized to the interval [0,1]. Note that scaling the inputs and outputs in this way requires a transformation of the PDE to the space of these new variables. Our network architecture and training strategy was based on the works of Raissi et all^16,17^ who published the original works outlining PINNs. In these works, various network structures and training methods were tested and analyzed. These choices in our work, unless otherwise noted, were made in accordance with their original recommendations. All code was run on an AMD EPYC 7542 2.9 GHz CPU and an NVIDIA Tesla A100 GPU. In the synthetic cases, each sample was optimized first over 30,000 Adam iterations, then the optimizer was switched to L-BFGS, and the training resumed until convergence (defined using a tolerance of 1.0e-8) or a maximum of 10,000 L-BFGS iterations was reached. In the animal data cases, the dual optimizer was again used, however training took place over 300,000 Adam iterations and a maximum of 30,000 L-BFGS iterations. Each synthetic case required approximately 10 minutes of run time, whereas each animal case required anywhere from 2-12 hours of run time, depending on its size. The drastic difference in training time between the synthetic and animal data cases is a result of the adherence of the data to the underlying PDE model. Specifically, in the synthetic case, the underlying data used to train the network matches the PDE closely (since the PDE was used to generate the data), and hence the PDE model is not an additional imposed assumption, making it amenable to training. Whereas in the case of the mouse data, the PDE model is a far rougher approximation of the experimental data, since it was generated *in vivo* (which in reality does not perfectly adhere to the PDE), meaning that the PDE is an additional imposed assumption, making it less amenable to training and take far longer to converge. This, in addition to the difference in total voxels due to the spatial dimension, results in significantly larger run times for the mouse data case compared to the synthetic data case.

## Results

### Predicting liposome accumulation and distribution

Using liposome accumulation maps derived from the imaging data, we implemented our deep learning model to perform voxel-by-voxel estimations of both liposome accumulation and IFP for each of the 10 mice included in our study. The model uses the final liposome accumulation map from imaging as known data and generates estimations of the temporal progression of liposome concentration as well as the spatial distribution of IFP within the tumour, both voxel-by-voxel. We consider the initial imaging to take place immediately prior to liposome administration, and therefore assume that the initial image is identically of zero magnitude. The parameters incorporated in equation (1) were assumed to correspond to the estimates provided in the original study by Stapleton et al^13^, which are detailed in Table 2.

In the study conducted by Stapleton et al^13^, each mouse was imaged at multiple time points. Using these time series data, we computed the corresponding liposome concentration maps for each time point. The neural network then utilized the final time point of each of these datasets, at 168 hours, to generate voxel-by-voxel prediction of liposome concentration at all times and IFP. Figure 3 presents a comparison of the measured and predicted liposome accumulations for each mouse, achieved in our predictions by calculating the mean voxel concentration within the tumour region.

Observe that for most of the mice considered in our study, the prediction of our deep learning model showed agreement with the observed results obtained through imaging. Some cases however, did not show good agreement. For example, the liposome accumulation comparison for mouse 08 shows a predicted peak concentration much higher than the observed peak concentration. Several factors could account for this discrepancy. First, since the network only uses data from a single time in order to make predictions, the time chosen to be used as known data can impact the results. In mouse 1 for example, the predicted liposome accumulation peak is higher than the observed peak; however, looking at the data points themselves, it is reasonable to question whether the final data point is an outlier as it appears out of step with the rest of the data. If a different time point had been selected to be used as known data, perhaps the error between the measured and predicted accumulation would have been less. Of course in practice, it would not be standard to conduct imaging at numerous times in this way, so one would not know whether an imaged time represented an outlier. Such is the nature of working with scarce, noisy data. Below, we compare our predictions for liposome accumulation and IFP generated using imaging from different times.

Another potential source of error comes from the selection parameters used in Eq. (1). In these simulations, the parameter values used are those in Table 2. In reality however, it would be reasonable to assume - and indeed, has been observed - that the tissue-specific parameters in the model can vary subject to subject. Changing the values of these parameters would impact the resulting predicted liposome accumulation curve and the prediction for IFP. Of course, obtaining estimates for the values of these parameters on a subject-by-subject basis is challenging, especially in pressure is also unknown. More discussion on this is provided in the conclusion section below.

### Predicting interstitial fluid pressure

In addition to the spatiotemporal accumulation of liposomes, our network also predicts the voxel-by-voxel IFP. Figure 4 shows a representative cross section of the intratumoral pressure for each of the mice in our study. Stapleton et al^13^ performed wick-in-needle pressure experiments to measure intratumoral IFP in 10 mice, though importantly, these 10 mice were a different 10 mice than were administered liposomes and imaged. This is because the wick-in-needle measurement method disrupts the liposome transport process, and therefore wouldn’t produce an accurate match of pressure and liposome distribution. However, the mice used for pressure measurements were otherwise identical to those used for imaging, so we can compare the sets of pressure measurements. In figure 5, a comparison of the measured and predicted average intratumoral IFP for the mice is shown. Each dot represents an individual mouse, and the dotted line represents the average of each case. Note that the interstitial pressure, $P_i$ must be lower than the microvascular pressure, $P_v=25$ mmHg, and therefore $P_v$ is the upper bound of the average intratumoral IFP. For the measured average IFP values, the minimum, maximum, and mean values were 13.63 mmHg, 24.18 mmHg, and 19.15 mmHg whereas for the predicted IFP average IFP values, they were 16.97 mmHg, 22.22 mmHg, and 20.77 mmHg.

Experimentally, the mean intratumoral pressure was calculated by performing 3 or 4 wick-in-needle measurements in the tumour volume, then calculating their mean, whereas here, the mean IFP was obtained by calculating the mean over every tumoral voxel. The averaging of a smaller number of measurements used to calculate the observed mean in^13^ could add additional error to the result, and given the small size of the tumour, may result in measurements made closer to the tumour boundary or slightly outside the tumour region, where the pressure tends to be lower. Furthermore, in^13^, it is noted that the pressure measurements themselves can cause a decrease in the tumour IFP, suggesting that perhaps multiple measurements could bias toward a slightly smaller result.

### Optimal imaging time for prediction of liposome accumulation and IFP

One key question is how the time of the chosen liposome accumulation image affects the predictions of the network. In other words, how long after injection of liposomes is required in order to derive an accurate estimation of IFP? In particular, our algorithm depends on the final time point image. However, our dataset comprises multiple time point images for each mouse, which allows for the flexibility to select any of these images as known data for optimizing our network. In this section, we do just this, assuming our final image to be each of those available from our dataset and comparing the network predictions. In each case, we derive the IFP and the liposome accumulation up to that time point, then use the PINN to project the concentration forward to the final available time, using the derived pressure to do so. This procedure allows us to analyze the discrepancies in predicted liposome accumulation and IFP when employing different time points as the basis for predictions. Due to the significant computational runtime associated with this process, we restricted our analysis to mouse 01 as a representative illustration. Figure 6 presents the results of this analysis.

On the left side of Figure 6, the predicted liposome accumulation curves based on the different chosen imaging time as known data is shown. Each of the curves passes through exactly two data points, the first (at time and concentration 0) and the point whose corresponding distribution is used as the final image. Several aspects should be noted here. First, note that the prediction resulting from using the first imaging time (10 minutes post injection) has been omitted from the graph since it’s height is significantly higher than the others, making the graph difficult to interpret for the remaining curves. Next, notice that the qualitative behaviour of the curve remains constant irrespective of the imaging time used; specifically, the time at which the maximal average concentration is reached remains constant. Additionally, as noted previously, notice that the final measured time point in the measured accumulation appears to be somewhat of an outlier compared to the general trend, which results in a predicted accumulation curve that does not align well with the rest of the data. In contrast, utilizing one of the intermediate time points as known data yields a curve that is closer to the remaining data points. Quantitatively, the error between the measured and predicted accumulation is shown in Table 4. Based on these residuals, the optimal time to image in this case is 72 hours post injection. Though any time between 11 and 120 hours appear to give reasonable fits to the remaining datapoints. Furthermore, the poor fit of the 144 and 164 hour predictions compared to the other datapoints suggests that these data points are outliers, matching our previous intuition. In practical scenarios, it is uncommon to conduct imaging at multiple time points like this, rendering it challenging to discern whether a given time point is likely to be an outlier. This challenge underscores the inherent complexity of working with noisy data, which may be difficult or costly to acquire. Though these results suggest that a range of times could be suitable for generating accurate predictions.

**Table 4:** Quantification of the error between the measured and predicted total accumulation from using different imaging times as known data. MSE is calculated by finding the sum of the squared errors between each measured accumulation and the predicted accumulation at that time.

Imaging Time (hours)	Prediction MSE
0.17	3024.31
11	2.85
12	3.11
24	3.30
48	2.91
72	2.77
98	3.82
120	3.18
144	8.18
164	12.01

On the right side of Figure 6, the prediction of average intratumoral IFP is shown. Importantly, the predictions are relatively stable with respect to the final imaging time. Excluding the initial measurement time of 10 minutes, all predicted IFP values fall within the range of [21.20 mmHg, 23.43 mmHg], which is easily explained by the choice of imaging time as known data. Based on this observation, we suggest that a single image, obtained one day post-administration, could suffice to generate reasonable predictions for both the complete liposome accumulation curve and the intratumoral IFP.

### Sensitivity analysis on synthetic tumours

For each parameter set, the deep learning network is run 5 times, and a bootstrapping algorithm with 1000 bootstrap samples is used to obtain the mean and 95% confidence interval estimate for the IFP prediction error. These can be seen in Fig. 7. Note that the model is able to approximate the error to within 3% error in all cases other than the maximum noise case. The highest error in the prediction of IFP (of about 6.5%) occurs in this high noise case, which is unsurprising since adding noise to the voxelwise concentration is expected to lead to errors in the model predictions. Similarly, the lowest error occurs when the noise is minimal. Of the remaining parameters, the predictions appear to be most sensitive to $\frac{L_pS}{V}$ and $P_v$, with larger values leading to larger errors for both. A relatively large error is also observed in the minimum $k_d$ case.

## Conclusion

In this work, we have developed a deep learning model for predicting intratumoral liposome accumulation and intratumoral IFP. The model utilizes the spatial distribution of liposome concentration throughout a tumour volume as input, which can be obtained through imaging. This approach has the potential to enable personalized predictions of the spatiotemporal distribution of cancer nanoparticles or other chemotherapeutic agents for individual patients. Additionally, IFP is a critical measure for tumours, with implications for disease progression and treatment response, knowledge of which could provide valuable information to clinicians. Conceptually, patients could receive an injectable dye, undergo imaging, and have the resulting data used in our model to predict key tumour characteristics. We applied our model to data derived from mouse xenograft tumour imaging and synthetically generated tumours. The inclusion of synthetic cases demonstrates the high accuracy of our model and robustness to measurement noise. Furthermore, the agreement between our model’s predictions and measured liposome accumulation in mouse experiments reinforces confidence in the model accuracy.

Our model offers several key advantages. Firstly, it requires minimal input data to make predictions, specifically a single image from a single patient. This contrasts with many machine learning models that require extensive image databases. Accordingly however, this necessitates the limitation of needing to retrain the model for each patient individually, which may incur significant computational costs. Another advantage is the model’s capability to handle measurement noise, a crucial attribute given the inherent errors present in medical imaging data. We demonstrated this by applying the model to synthetic tumours with added noise and observing that a 5% noise added voxelwise to our known data resulted in an average error in the prediction of pressure of only approximately 6.5%. Similar results have been reported in previous studies using physics-informed neural networks (PINNs)^16,17,21^. The voxel-by-voxel prediction capability also allows for spatial predictions of drug distribution and improved understanding of individual tumours.

Despite these advantages, there are areas for improvement. Direct comparisons of predicted to measured IFP is not possible in our work, as pressure measurements using a wick-and-needle technique can affect liposome transport itself, compromising the accuracy of liposome accumulation data for network training. Theoretically, liposome imaging could precede pressure measurements or additional interventions, but the nature of our data did not support this. Instead, the data in our study used two separate sets of mice, by which we can compare the two groups to see the differences in IFPs, but are unable to compare for particular mice. More generally, the question of validation in our model is challenging since it trains on an individual subject’s data exclusively. The techniques used within for validation do not provide a true validation, since there is no meaningful split of the available data into training, validation, and testing sets. Rather, the inclusion of comparison of our animal model predictions to bulk liposome accumulation at intermediate times and an equivalent distribution of average intratumoral pressures provides a pseudo-validation. Similarly, the application of our model to synthetic tumours allows for a validation of the mathematical and computational model in general, and when applied to idealized data, but does not necessarily provide validation for its use on *in vivo* data. Additionally, our model does not account for liposomes outside the tumour volume, which are expected to be present in reality. Though since we are primarily interested in intratumoral IFP, we don’t expect the small amount of particles outside the tumour volume to have a large effect on the derivation of intratumoral IFP.

Our largest limitation to clinical translation however, is the difficulty of measuring the parameters of the liposome transport model (Eq. 1). Ideally, we would like to estimate both the parameter values and IFP simultaneously. While PINNs can theoretically achieve this, the challenge lies in uniquely identifying these values given available information. In particular, in Eq. 1, $\frac{L_pS}{V}$ and $(P_v - P_i)$ are multiplied together, allowing only their product to be uniquely identified. Accordingly, we are forced to either fix the parameter values and derive the IFP, or fix the IFP and derive the parameter values. In our study, we chose to fix the parameter values using the estimates made by Stapleton et al^13^, and derive the IFP. This data offered an opportunity since the parameters were estimated for the specific set of mice and contained measurements of intratumoral IFP to which we could compare our predictions. In the absence of these parameter estimates, representative values from the literature could be used, or a range of parameter values could be sampled and used in the optimization.

Alternatively, there may be a path toward estimating both the parameter values simultaneously; however, more information than we considered would need to be included in order to do this. For example, the optimization method could use all of the imaging time points together in order to make a prediction, rather than just an initial and final image. Though inherent difficulties exist in this too. First, in practice a series of images is unlikely to be performed, so this method would not scale well. And second, using many images would require that these images were accurately coregistered together, which is theoretically possible in cases where sophisticated registration algorithms exist (like in the context of brain tumours), though very challenging in situations where they do not. Another potential avenue to estimate both the parameters and pressure simultaneously is through the incorporation of additional mathematics into the optimization. Specifically, consider the idea that the PDE for pressure, equation (8), is included in the optimization. Then, the deep learning model could produce estimates for the unknown parameters of interest, use these values to predict IFP using Eq. (8), then use the parameters and IFP to calculate the loss. This idea has potential downsides as well however. For one, doing so requires that an additional PDE is solved at each optimization iteration, adding a significant amount of computational expense. Furthermore, this optimization is conceptually different since the network would no longer be predicting the pressure directly, but rather, predicting the parameters, then using them to derive the pressure. Since the parameters are assumed to be constant within the volume of the tumour, this would force the network to lose some of the voxel-by-voxel specificity in predictions of intratumoral IFP.

Similarly, our model also relies on the assumption of a spatially-uniform plasma concentration throughout the tumour volume, which aligns with the approach employed by Stapleton et al.^13^ in their analysis. Essentially, this assumption implies that variations in the imaged distribution of liposomes within the tumour can be solely attributed to underlying spatial variations in IFP, rather than the plasma concentration per voxel. In reality however, both the IFP and plasma concentration per voxel are expected to vary throughout the tumour volume, and therefore impact the resulting spatially-varying accumulation. This is especially true considering that both high IFP and impaired plasma concentration are effects of an underlying dysfunctional tumour vasculature. More accurately, we expect the plasma concentration of liposomes to be approximately uniform throughout the vessels, but for the vessel density itself to vary throughout the tumour. Similar to the challenge posed by unknown parameter values described above, without additional data or mathematical assumptions, it is necessary to fix one variable to uniquely predict the other. Given the experimental data available to us, we have opted to fix the plasma concentration and predict the pressure.

Though in theory, it is possible to separate the effects of IFP and plasma concentration for a given subject. However, accomplishing this would require either 1) obtaining more data or 2) incorporating additional mathematical assumptions. For more data, if vessel perfusion imaging were available alongside liposome accumulation imaging for our subjects, it could be used to derive a spatial distribution of plasma concentration. This information could then be incorporated into our model, allowing for the decoupling of the effects of IFP and plasma concentration. Other types of data could also allow for a similar separation of these effects. For instance, if liposome accumulation data were available for multiple dyes within the same subject, it could be possible to mathematically decouple the two fields. The different transport parameters (such as hydraulic conductivity or diffusivity) associated with each dye could be utilized to mathematically separate the effects of these processes. Similarly, the application of antiangiogenic agents before liposome administration could allow for the observation of changes in liposome accumulation under different tumour vasculatures induced by the antiangiogenics, thus similarly enabling the separation of IFP and vasculature effects. For incorporating more mathematics, a different set of model equations could be assumed, coupling both the IFP and the plasma concentration to an underlying vascular density. Then, in theory, the PINN could be used to predict this underlying vasculature and the IFP and plasma concentration could be derived from it. However, preliminary tests have shown that this process incurs substantial computational expense, making it infeasible at present. Nonetheless, it represents a potential path forward for simultaneous prediction, provided computational challenges are overcome.

In summary, this exploratory study presents a novel machine learning approach to predict liposome accumulation and IFP from imaging data, advancing personalized medicine. We hope this work showcases the potential benefits of deep learning in oncology and medical imaging, offering clinicians a powerful tool for forecasting tumour progression, designing effective treatments, and predicting treatment efficacy.

## Data Availability

Relevant data and code for this publication is available online at https://github.com/cfmeaney/Lipo_IFP_PINN.
